# Assessing Full Benefit of Rivaroxaban Prophylaxis in High-Risk Ambulatory Patients with Cancer: Thromboembolic Events in the Randomized CASSINI Trial

**DOI:** 10.1055/s-0040-1712143

**Published:** 2020-05-23

**Authors:** Alok A. Khorana, Mairéad G. McNamara, Ajay K. Kakkar, Michael B. Streiff, Hanno Riess, Ujjwala Vijapurkar, Simrati Kaul, Peter Wildgoose, Gerald A. Soff, on behalf of the CASSINI Investigators

**Affiliations:** 1 Department of Hematology and Medical Oncology, Cleveland Clinic, Cleveland, Ohio, United States; 2 Division of Cancer Sciences, Department of Medical Oncology, University of Manchester, The Christie NHS Foundation Trust, Manchester, United Kingdom; 3 Thrombosis Research Institute and University College London, London, United Kingdom; 4 Division of Hematology, Department of Medicine, Johns Hopkins University School of Medicine, Baltimore, Maryland, United States; 5 Department of Medicine, Charité Universitätsmedizin Berlin, Berlin, Germany; 6 Clinical Biostatistics, Janssen Research & Development, LLC, Raritan, New Jersey, United States; 7 Medical Affairs Internal Medicine, Janssen Scientific Affairs, LLC, Titusville, New Jersey, United States; 8 Hematology Service, Memorial Sloan Kettering Cancer Center, New York, New York, United States

**Keywords:** arterial thrombosis, cancer, thrombolysis/thrombolytic agents, thromboprophylaxis, venous thrombosis

## Abstract

**Introduction** In the CASSINI study, rivaroxaban thromboprophylaxis significantly reduced primary venous thromboembolism (VTE) endpoints during the intervention period, but several thromboembolic events designated as secondary efficacy endpoints were not included in the primary analysis. This study was aimed to evaluate the full impact of rivaroxaban thromboprophylaxis on all prespecified thromboembolic endpoints occurring on study.

**Methods** CASSINI was a double-blind, randomized, placebo-controlled study in adult ambulatory patients with cancer at risk for VTE (Khorana score ≥2). Patients were screened at baseline for deep-vein thrombosis (DVT) and randomized if none was found. The primary efficacy endpoint was a composite of lower extremity proximal DVT, symptomatic upper extremity, or lower extremity distal DVT, any pulmonary embolism, and VTE-related death. This analysis evaluated all prespecified thromboembolic endpoints occurring on study to determine the full benefit of rivaroxaban prophylaxis. All endpoints were independently adjudicated.

**Results** Total thromboembolic events occurred in fewer patients randomized to rivaroxaban during the full study period (29/420 [6.9%] and 49/421 [11.6%] patients in rivaroxaban and placebo groups, respectively [hazard ratio (HR) = 0.57; 95% confidence interval (CI): 0.36–0.90; *p* = 0.01]; number needed to treat [NNT] = 21). Similarly, fewer patients randomized to rivaroxaban experienced thromboembolism during the intervention period (13/420 [3.1%] patients) versus placebo (38/421 [9.0%] patients; HR = 0.33; 95% CI: 0.18–0.62; *p* < 0.001; NNT = 17).

**Conclusion** Our findings confirm the substantial benefit of rivaroxaban thromboprophylaxis when considering all prespecified thromboembolic events, even after excluding baseline screen-detected DVT. The low NNT, coupled with prior data demonstrating a high number needed to harm, should assist clinicians in determining the risk/benefit of thromboprophylaxis in high-risk patients with cancer.

## Introduction

Venous and arterial thromboembolism frequently complicate the natural history of cancer and cancer-directed treatment. 1 2 Given that cancer therapy is predominantly delivered in the outpatient setting, thromboembolism is also most likely to occur there. Prior trials of both low–molecular weight heparins and, recently, direct oral anticoagulants (DOACs) have evaluated the benefit of thromboprophylaxis in outpatients with cancer receiving systemic therapy. 3 4 5 6

We have previously reported the primary analysis results of one such randomized trial of outpatient thromboprophylaxis with daily rivaroxaban in ambulatory patients with cancer at higher risk for venous thromboembolism (VTE), based on a Khorana score of 2 or higher. 5 In this analysis of 841 randomized patients, the primary endpoint was a composite of any objectively confirmed symptomatic or asymptomatic lower extremity proximal deep-vein thrombosis (DVT), symptomatic upper extremity DVT, symptomatic lower extremity distal DVT, any pulmonary embolism (PE), or VTE-related death. The primary endpoint occurred in 6.0% of patients in the rivaroxaban group, compared with 8.8% in the placebo group during the up-to-day-180 period analysis and in 2.6% of patients in the rivaroxaban group and 6.4% in the placebo group in the intervention-period analysis. However, additional prespecified thromboembolic endpoints were collected including arterial events (ischemic stroke, myocardial infarction, or peripheral arterial thromboembolism), screen-detected distal DVT, and visceral vein thromboembolism. All of these events were adjudicated by a blinded committee but not included in the primary analysis. The totality of benefit of rivaroxaban thromboprophylaxis can be clarified by evaluating its impact on all of these thromboembolic events, in addition to the primary endpoint events.

We therefore conducted this analysis of the CASSINI study to assess the full benefit of rivaroxaban thromboprophylaxis by combining all prespecified thromboembolic endpoints in the population of all randomized patients for both the full study period and the intervention period.

## Methods

CASSINI was a phase 3b, randomized, double-blind, placebo-controlled, parallel-group, and multicenter trial. The study design for CASSINI has previously been described. 5 7 Briefly, we included adult ambulatory (outpatients) with a solid tumor or lymphoma, locally advanced or metastatic disease, baseline Khorana score of 2 or higher, and an expected survival of greater than 6 months, with a plan to start a new systemic regimen within 1 week of initiating study drug. Patients with primary brain tumors or known brain metastases, Eastern Cooperative Oncology Group (ECOG) performance status of 3 or higher, active bleeding, or at risk for bleeding were excluded. Patients without DVT on baseline screening were randomized 1:1 to rivaroxaban (XARELTO; Janssen) 10 mg or placebo orally once daily for 180 (±3) days.

The primary efficacy endpoint measure was the composite of objectively confirmed symptomatic or asymptomatic lower extremity proximal DVT, symptomatic upper extremity or lower extremity distal DVT, symptomatic or incidental PE, and venous thromboembolism–related death, as adjudicated by an independent blinded clinical endpoint committee. Secondary efficacy endpoint measures included components of the primary endpoint, including symptomatic venous thromboembolism, as well as clinically relevant events not included in the primary composite endpoint, such as all-cause mortality, confirmed arterial thromboembolism, and confirmed visceral thromboembolism. The study was performed in accordance with the Declaration of Helsinki and local regulations. The protocol was approved by institutional review boards at each study site.

For this analysis, the total thromboembolic endpoint was defined to determine the full benefit of thromboprophylaxis. The total thromboembolic endpoint included all components of the primary efficacy endpoint (symptomatic lower extremity proximal DVT, symptomatic lower extremity distal DVT, symptomatic upper extremity DVT, asymptomatic lower extremity proximal DVT, symptomatic nonfatal PE, incidental PE, and VTE-related death), as well as confirmed arterial thromboembolism (myocardial infarction, ischemic stroke, and systemic arterial embolism), visceral thromboembolism, and asymptomatic (screen detected) lower extremity distal DVT.

### Statistical Analysis

The analysis of the total thromboembolic endpoint was based on the intent-to-treat (ITT) analysis population, which comprised all of the patients who had undergone randomization, with data from randomization through day 180. In addition, analysis during the intervention period (from start of treatment through end of treatment + 2 days) was also performed.

Cumulative event rates for the total thrombotic composite endpoint were estimated with the use of the Kaplan–Meier method, and the *p* -value was calculated by the two-sided log-rank test, stratified according to tumor type. The total thrombotic endpoint was analyzed with the use of a stratified Cox proportional hazards model, with study treatment as a covariate and tumor type (pancreatic vs. not pancreatic) as a stratification factor, to provide a point estimate (hazard ratio) and 95% confidence interval (CI). No adjustments for multiple comparisons were made for the additional endpoint for this analysis. All statistical analyses were performed with SAS software, version 9.4 (SAS Institute, Cary, NC).

## Results

The study population comprised all 841 randomized patients. During the full study period, events in addition to the primary endpoint included arterial thromboembolism, which occurred in 4 of 420 patients (1.0%) in the rivaroxaban arm and 7 of 421 patients (1.7%) in the placebo arm ( Table 1 ). Screen-detected distal DVT occurred in 1 of 420 patients (0.2%) in the rivaroxaban arm and 6 of 421 patients (1.4%) in the placebo arm. Visceral vein thrombi occurred in 1 of 420 patients (0.2%) in the rivaroxaban arm and 2 of 421 patients (0.5%) in the placebo arm. During the intervention period, events in addition to the primary endpoint events included arterial thromboembolism, which occurred in 0.5% of patients in the rivaroxaban arm and 1.2% of patients in the placebo arm; screen-detected distal DVT in 0% of patients in the rivaroxaban arm and 1.2% of patients in the placebo arm; and visceral vein thrombi, which occurred in 0% of patients in the rivaroxaban arm and 0.5% of patients in the placebo arm ( Table 2 ).

**Table 1:** Primary and secondary thromboembolic endpoint events during the full study period, according to study group 5

	Rivaroxaban ( *n* = 420) % ( *n* )	Placebo ( *n* = 421) % ( *n* )	HR (95% CI)	*p* -Value a
Primary endpoint events b	6.0 (25)	8.8 (37)	0.66 (0.40–1.09)	0.101
Symptomatic event	3.6 (15)	4.5 (19)	–	
Symptomatic proximal DVT in lower limb	2.1 (9)	1.9 (8)	1.12 (0.43–2.91)	
Symptomatic distal DVT in lower limb	0.5 (2)	1.2 (5)	0.40 (0.08–2.07)	
Symptomatic DVT in upper limb	1.0 (4)	1.4 (6)	0.67 (0.19–2.39)	
Symptomatic nonfatal PE	1.2 (5)	1.2 (5)	1.02 (0.29–3.52)	
Asymptomatic event	2.1 (9)	4.3 (18)	–	
Asymptomatic proximal DVT in lower limb	1.0 (4)	2.6 (11)	0.35 (0.11–1.11)	
Incidental PE	1.4 (6)	2.4 (10)	0.59 (0.21–1.62)	
VTE-related death	0.2 (1)	0.7 (3)	0.33 (0.03–3.18)	
Other thromboembolic events				
Arterial c	1.0 (4)	1.7 (7)	0.58 (0.17–1.98)	
Visceral d	0.2 (1)	0.5 (2)	0.51 (0.05–5.58)	
Screen-detected distal DVT	0.2 (1)	1.4 (6)	0.15 (0.02–1.29)	
Total thromboembolic events	6.9 (29)	11.6 (49)	0.57 (0.36–0.90)	0.014

Abbreviations: CI, confidence interval; DVT, deep-vein thrombosis; HR, hazard ratio; PE, pulmonary embolism; VTE, venous thromboembolism.

a *p* -Value for the primary endpoint was based on log-rank test, stratified by tumor type (advanced pancreatic cancer vs. not). *p* -Values for secondary and other endpoints were not adjusted for multiple comparisons.

b Primary efficacy composite endpoint: time from randomization to first occurrence of objectively confirmed symptomatic lower extremity proximal DVT, asymptomatic lower extremity proximal DVT, symptomatic lower extremity distal DVT, symptomatic upper extremity DVT, symptomatic nonfatal PE, incidental PE, or VTE-related death.

c A composite of occurrence of myocardial infarction, stroke (ischemic infarction with or without hemorrhagic conversion or primary hemorrhagic events [e.g., intraparenchymal hemorrhage, subdural hematoma, or epidural hematoma]), or any other arterial thromboembolic event.

d Fatal/nonfatal visceral VTE events.

**Table 2:** Primary and secondary thromboembolic endpoint events during the intervention period, according to study group 5

	Rivaroxaban ( *n* = 420) % ( *n* )	Placebo ( *n* = 421) % ( *n* )	HR (95% CI)	*p* -Value a
Primary endpoint events b	2.6 (11)	6.4 (27)	0.40 (0.20–0.80)	0.007
Symptomatic event	1.2 (5)	2.9 (12)	–	
Symptomatic proximal DVT in lower limb	0.7 (3)	1.0 (4)	0.72 (0.16–3.22)	
Symptomatic distal DVT in lower limb	0	0.5 (2)	NA	
Symptomatic DVT in upper limb	0.5 (2)	1.4 (6)	0.33 (0.07–1.63)	
Symptomatic nonfatal PE	0.2 (1)	0	NA	
Asymptomatic event	1.2 (5)	3.6 (15)	–	
Asymptomatic proximal DVT in lower limb	0.7 (3)	2.4 (10)	0.29 (0.08–1.07)	
Incidental PE	0.5 (2)	1.2 (5)	0.38 (0.07–1.98)	
VTE-related death	0.2 (1)	0.2 (1)	0.97 (0.06–15.55)	
Other thromboembolic events				
Arterial c	0.5 (2)	1.2 (5)	0.39 (0.08–2.03)	
Visceral d	0	0.5 (2)	NA	
Screen-detected distal DVT	0	1.2 (5)	NA	
Total thromboembolic events	3.1 (13)	9.0 (38)	0.33 (0.18–0.62)	<0.001

Abbreviations: CI, confidence interval; DVT, deep-vein thrombosis; HR, hazard ratio; NA, not available; PE, pulmonary embolism; VTE, venous thromboembolism.

a *p* -Value for the primary endpoint was based on log-rank test, stratified by tumor type (advanced pancreatic cancer vs not). *p* -Values for secondary and other endpoints were not adjusted for multiple comparisons.

b Primary efficacy composite endpoint: time from dose start date to first occurrence of objectively confirmed symptomatic lower extremity proximal DVT, asymptomatic lower extremity proximal DVT, symptomatic lower extremity distal DVT, symptomatic upper extremity DVT, symptomatic nonfatal PE, incidental PE, or VTE-related death.

c A composite of occurrence of myocardial infarction, stroke (ischemic infarction with or without hemorrhagic conversion or primary hemorrhagic events [e.g., intraparenchymal hemorrhage, subdural hematoma, or epidural hematoma]), or any other arterial thromboembolic event.

d Fatal/nonfatal visceral VTE events.

**Fig. 1:**
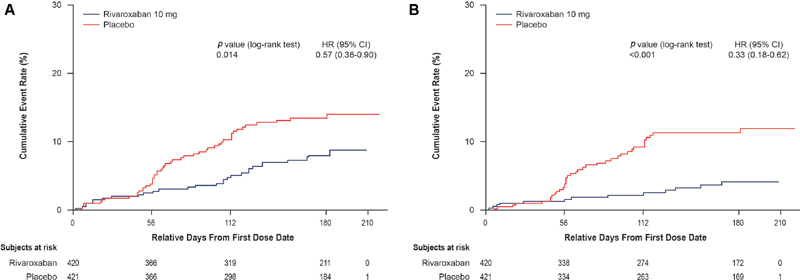
Kaplan–Meier curves for all thromboembolic outcomes in all randomized patients, according to study group. Primary and secondary endpoints together included objectively confirmed, symptomatic, lower extremity, proximal or distal DVT; asymptomatic, lower extremity, proximal DVT; symptomatic upper extremity DVT; symptomatic or incidental nonfatal PE; or venous thromboembolism–related death; confirmed arterial thromboembolism; confirmed visceral thromboembolism, and confirmed distal asymptomatic DVT during the up-to-day 180 period ( **A** ): HR = 0.57; 95% CI: 0.36–0.90; *p* = 0.01 or the intervention period ( **B** ); HR = 0.33; 95% CI: 0.18–0.62; *p* < 0.001. Every patient was accounted for in the analysis of the primary efficacy composite endpoint for the intervention period. An imputation rule using the time patient in the double-blind period from randomization was implemented for patients who had never dosed to ensure patients were not excluded from on-treatment analysis for the ITT population. CI, confidence interval; DVT, deep-vein thrombosis; HR, hazard ratio; ITT, intent to treat; PE, pulmonary embolism.

When primary and secondary efficacy endpoints were combined, total thromboembolic events occurred in fewer patients randomized to rivaroxaban during the full study period (total thromboembolic events in 29/420 [6.9%] and 49/421 [11.6%] patients in rivaroxaban and placebo groups, respectively; hazard ratio [HR] = 0.57; 95% CI: 0.36–0.90; *p* = 0.01; number needed to treat [NNT] = 21; Fig. 1A ). Of these 78 events, 27 (34.6%) occurred after drug discontinuation. Similarly, fewer patients randomized to rivaroxaban experienced thromboembolic events during the intervention period (13/420 [3.1%] patients) versus placebo (38/421 [9.0%] patients; HR = 0.33; 95% CI: 0.18–0.62; *p* < 0.001; NNT = 17; Fig. 1B ).

## Discussion

We report our analysis of the full benefit of rivaroxaban thromboprophylaxis in ambulatory patients with cancer receiving systemic therapy when evaluating all prespecified and adjudicated thromboembolic endpoints. Our findings demonstrate significant reductions in thromboembolic events with the use of rivaroxaban and support the findings of the primary analysis suggesting benefit for patients with this approach.

The rates of thromboembolic events observed in this population, selected after ultrasonographic screening for DVT, are substantial, 11.6% in the placebo group during the 180-day study period. If prerandomization screening had not been conducted, it is likely that a proportion of the 4.5% of patients with screen-detected DVT would have developed subsequent symptomatic events, further adding to the thromboembolic burden observed in this population. 8 In a retrospective analysis of 300 patients with asymptomatic lower extremity DVT (70% with distal DVT only), 17 developed symptomatic recurrent VTE during the 5-year follow-up; however, active cancer was present in 8 of these 17 patients, indicating a higher risk for developing symptomatic VTE in cancer patients. 9 Anticoagulant therapy in the cancer subgroup was associated with a reduced risk of symptomatic VTE. In the CASSINI trial, patients underwent duplex compression ultrasonography (CU) during screening, at weeks 8 and 16, and at the end of treatment. This evaluation was an important feature of this study because it allowed detection of asymptomatic VTE over the course of the study. Although symptomatic disease is most important, many cases of DVT and PE are asymptomatic. In a 2010 study, asymptomatic DVT was found in 34% of nonambulatory cancer patients, with 11% of DVT in proximal veins and 23% in distal veins. 10 In another study, symptomatic and asymptomatic VTE occurred in 35% of patients with pancreatic cancer, and both were associated with mortality. 11 Gary and colleagues found asymptomatic VTE in the lower limbs of 18% of patients with cancer and these thromboembolic events were associated with a 2.4-fold risk of death during 9 months of follow-up independent of cancer stage, tumor type, and therapy for cancer. 12 Asymptomatic DVT identified by screening represents a subclinical manifestation of disease, and recommendations for prophylaxis are largely based on studies that used such routine screening approaches. Thus, the absolute risk reduction observed (4.7% during the full study period and nearly 6% during the intervention period) is clinically meaningful and suggests that the primary analysis underestimates the benefit of thromboprophylaxis in higher risk patients with cancer.

Our findings are consistent with a prior large randomized trial of nadroparin prophylaxis in mixed cancer populations, Prophylaxis of Thromboembolism during Chemotherapy (PROTECHT), which used a similarly defined on-treatment period and included arterial events for primary analysis. 3 However, the absolute risk reduction is tripled (6% compared with 1.9% for PROTECHT). Similarly, high–absolute risk reduction was observed in the A Very Early Rehabilitation Trial after stroke (AVERT) study, a comparable trial using a different DOAC, apixaban. 6 AVERT did not report arterial thromboembolism endpoints, so our current analysis of CASSINI provides the only opportunity to study the benefit of thromboprophylaxis for arterial events in this population. Arterial and isolated distal thromboembolism have clinically important consequences for patients with cancer. 13 14 Although we included visceral events in evaluating our total thromboembolic endpoint, these only accounted for a very small number of events. Taking all of these events into account, the NNT to prevent one thromboembolic event was 17.

Bleeding is, of course, an important consideration to balance against the benefit of thromboprophylaxis. We have previously published major and clinically relevant nonmajor bleeding rates in CASSINI. 5 Major bleeding occurred in 2.0% of patients in the rivaroxaban group and in 1.0% of the placebo group (HR = 1.96; 95% CI: 0.59–6.49; number needed to harm [NNH] = 101). Clinically relevant nonmajor bleeding occurred in 2.7% of patients in the rivaroxaban group compared with 2.0% of those in the placebo group (HR = 1.34; 95% CI: 0.54–3.32; NNH = 135). Careful consideration of bleeding risk is important when evaluating the risk/benefit balance of thromboprophylaxis in individual patients.

## Limitations

There are certainly limitations to this analysis. This represents a post hoc analysis of a prospective randomized trial. However, all thromboembolic endpoints included in the total rate were prespecified and blindly adjudicated. Additionally, we have chosen to analyze the ITT population (i.e., all randomized patients), including those patients who never received drug, to be conservative rather than conduct a modified ITT or per-protocol analysis, which could overestimate the full benefit of thromboprophylaxis.

## Conclusion

In conclusion, our findings confirm the substantial benefit of rivaroxaban thromboprophylaxis when considering all prespecified thrombotic events. The low NNT reported here, coupled with our prior data demonstrating a high NNH for major and clinically relevant nonmajor bleeding, should better clarify the net benefit of prophylaxis and assist clinicians in clinical decision making regarding thromboprophylaxis in this high-risk population.
